# Challenges on non-invasive ventilation to treat acute respiratory failure in the elderly

**DOI:** 10.1186/s12890-016-0310-5

**Published:** 2016-11-15

**Authors:** Raffaele Scala

**Affiliations:** Pulmonology and RICU, S. Donato Hospital, Arezzo, Italy

**Keywords:** Acute respiratory failure, Non-invasive mechanical ventilation, Endotracheal intubation, End-of-life, High-flow nasal cannula, CO2-removal, Bronchoscopy, Elderly patient

## Abstract

Acute respiratory failure is a frequent complication in elderly patients especially if suffering from chronic cardio-pulmonary diseases. Non-invasive mechanical ventilation constitutes a successful therapeutic tool in the elderly as, like in younger patients, it is able to prevent endotracheal intubation in a wide range of acute conditions; moreover, this ventilator technique is largely applied in the elderly in whom invasive mechanical ventilation is considered not appropriated. Furthermore, the integration of new technological devices, ethical issues and environment of treatment are still largely debated in the treatment of acute respiratory failure in the elderly.

This review aims at reporting and critically analyzing the peculiarities in the management of acute respiratory failure in elderly people, the role of noninvasive mechanical ventilation, the potential advantages of applying alternative or integrated therapeutic tools (i.e. high-flow nasal cannula oxygen therapy, non-invasive and invasive cough assist devices and low-flow carbon-dioxide extracorporeal systems), drawbacks in physician’s communication and “end of life” decisions. As several areas of this topic are not supported by evidence-based data, this report takes in account also “real-life” data as well as author’s experience.

The choice of the setting and of the timing of non-invasive mechanical ventilation in elderly people with advanced cardiopulmonary disease should be carefully evaluated together with the chance of using integrated or alternative supportive devices. Last but not least, economic and ethical issues may often challenges the behavior of the physicians towards elderly people who are hospitalized for acute respiratory failure at the end stage of their cardiopulmonary and neoplastic diseases.

## Background

Thanks to the modern pharmacological and non-pharmacological approach (i.e.*,* long-term oxygen therapy and home mechanical ventilation) pulmonologists are able to prolong the survival of patients with chronic respiratory diseases till the very advances stages of their natural history [1]. However, the increase in the survival rate is not always associated with a satisfactory quality of life since an impaired lung function is often associated with a limitation in his/her daily activities living and with discomfort (i.e.*,* dyspnea, weakness, depressive symptoms) [2, 3]. As a matter of the fact, prolonging survival is not always a desirable goal to achieve for both the physician and the patient according to the modern vision of patient-centred management of diseases [4]. Conversely, palliation of symptoms and shared “end-of-life” decisions are the main target of the care in order to keep a human dignity in the transition to the death in patients with advanced chronic respiratory diseases [5]. On the other hand, severely disabled patients with “end-stage” cardio-pulmonary disease may agree to undergo life-support devices (i.e. mechanical ventilation) in Hospital to overcome an episode of acute decompensation even if the impairment in their quality of life at home may still progress depending on the severity of their underlying condition. Moreover, undue “nihilism” about outcomes of invasive mechanical ventilation (IMV) in conditions such as chronic obstructive lung disease (COPD), is common and of concern, as patients may be swayed by inaccurate inferences about the likely outcome, should support be provided. Because decisions on whether to admit patients with COPD or asthma to intensive care setting for endotracheal intubation (ETI) depend on clinicians’ prognoses, the CAOS study [6] highlighted that in the some patients who might otherwise survive are probably being denied admission because of unwarranted prognostic pessimism of physicians. Within this context, the management of patients at the “end of stage” of their underlying cardiopulmonary disease who are hospitalised for an episode of acute respiratory failure (ARF) may raise ethical dilemma concerning the choice of the level of the “escalating therapy” that could be potentially offered ranging from the medical and oxygen-therapy till IMV [4].

With the progressive extension of the natural history of many chronic pulmonary and extra-pulmonary diseases, inevitably the incidence rate of elderly patients admitted in the hospital for ARF is continuously increasing. Accordingly, the risk of developing a boot of ARF increases with the age and this phenomenon occurs mainly in elderly people with underlying chronic cardio-pulmonary disorders, immunosoppressed conditions, solid and hematologic malignancies and coexisting multiple extra-pulmonary comorbidities [7]. Triggering causes of ARF in advanced aged patients are especially acute heart decompensation, severe community acquired pneumonia, acute exacerbations of COPD, drug-induced lung injury. In a subset of elderly patients the deterioration of lung function may occur without any evidence of a superimposed condition and could be considered as an inevitably progression of the natural history of the underlying disease [7].

## Main text

### Therapeutic options for acute respiratory failure in the elderly

Similarly to the adult population, the first support for the treatment of ARF in the elderly is represented by the delivery of conventional oxygen therapy via a facemask or nasal cannula to “buy the time” for the etiologic therapy to reverse the cause of the acute decompensation of the respiratory system [8, 9]. Once physicians realize that only oxygen-therapy is not enough to properly and quickly correct the impaired lung gas exchange and to reduce the burden of respiratory distress, non-invasive ventilation (NIV) becomes the following option whose aim is to avoid the need for IMV, as well as to prevent its life-threatening complications [10]. Even though in the last two decades NIV has dramatically changed the epidemiology of mechanical ventilation in an expanding number of acute clinical scenarios, the chance of success with this ventilatory technique is variable and strongly depends on several variables, such as team’s experience, patient-ventilator synchrony, air leaks, adequate equipment and environment, patho-physiology pattern, timing and severity of ARF [10]. Above all, the adherence to the scheduled ventilatory treatment is the crucial “ingredient” for successfully adapting, carrying on and weaning the patient off from the ventilator [10–12]. As a matter of the fact, when NIV is or become a unviable therapeutic option, the subsequent mandatory option is ETI and IMV delivered in high-intensity monitored hospital setting. Furthermore, refractory hypoxemia during IMV in severe acute respiratory distress syndrome (ARDS) may require the implementation of “artificial lung” by means of extra-corporeal membrane oxygenation (ECMO) [13]. At this stage of ARF, multiple organ dysfunction is likely to occur with the need for extra-pulmonary support, such as renal replacement therapy.

In case of refusal of life-support devices, palliation care and terminal sedation is an “end-of-life” therapeutic option which may be considered by both the physician and the patient [4]. New therapeutic options could be applied as either alternative or integrative supportive strategy to NIV, such as high-flow nasal cannula (HFNC) [9], non-invasive cough assist devices -such as mechanical insufflator-exsufflator (MI-E) [14], high frequency chest wall oscillation (HFCWO) [15], flexible bronchoscopy (FOB) [16], extracorporeal CO_2_ removal (ECCO_2_R) [17] both in patients who are failing NIV and in those who are at risk of developing extubation failure.

### Mechanical ventilation in the elderly

**Fig. 1:**
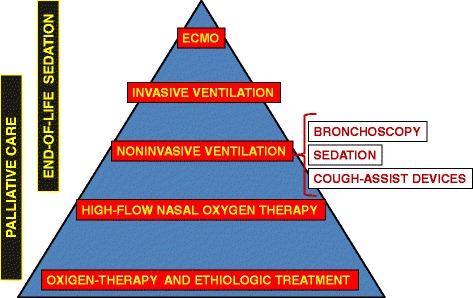
Therapeutic options in elderly with acute respiratory failure

While the application of the conservative therapeutic approach based on the solely administration of drugs and oxygen within the context of non-intensive care setting (i.e.*,* ward and other un-monitored areas) is not dissimilar in the elderly than in adults, the choice of administering mechanical ventilation in higher intensity of care environment [i.e.*,* general Intensive Care Units (ICU) and Respiratory Intensive Care Units (RICU)] in octogenarians is controversial [7]. In fact, despite the prognosis of patients undergoing mechanical ventilation has not been shown to be different in the elderly than in the younger population, the former usually receive less intensive and expensive care than the latter having the same degree of clinical-physiological derangement. This is particularly evident in the elderly with chronic cardio-pulmonary diseases hospitalized for an episode of ARF for whom the denial of ICU/RICU admission is often determined by an unjustified pessimistic prognostic perspective shown by physicians [6]. The still poorly defined border between “curative”, “palliative” and “end-of-life” treatment in patients with “end-stage” lung diseases makes more complex this clinical scenario [4, 5, 18]. In patients with advanced respiratory disease, individual Hospital units vary greatly in regard to selection based on age. Even if the “age-based” restriction access to higher level of care is not justified by the existing data, in some institutions the label of “do-not-intubate” order (DNI) may be applied to octogenarians with chronic advanced respiratory disease [4]. In the Fig. 1 the therapeutic options available in the elderly with ARF are depicted.

### The role of non-invasive ventilation in the elderly

#### Rationale

NIV is the first-choice ventilatory technique in some diseases (COPD, cardiogenic pulmonary edema, immunosuppression of different origin, neuromuscular disease without severe bulbar impairment, obesity hypoventilation syndrome and chest wall deformity) which have an high prevalence in the elderly [10]. The advantage of NIV is to offer the same physiological effects of IMV delivered via ETI (i.e.*,* unloading respiratory muscles, improvement gas exchange, augment alveolar ventilation) avoiding the risks correlated with the use of an artificial airway (endotracheal tube, tracheostomy cannula), such as ventilator-associated pneumonia, whose incidence rate is especially high in the elderly [19]. Since most patients receiving NIV are managed without sedation, early weaning off from the ventilator is facilitated and sedative drug-related complications are avoided. Keeping in mind the drawbacks of NIV (i.e.*,* lack of airway protection, need of patient’s cooperation, preserved cough reflex), the early use of this ventilatory technique in addition to the oxygen and medical therapy for the management of ARF is able to significantly reduce the rate of intubation, the length of hospital stay and the hospital mortality rate especially in acidotic hypercapnic patients, included those in their elderly age [10]. In addition to that, it has been documented the effectiveness of NIV as “ceiling ventilatory treatment” in DNI elderly patients with chronic cardio-respiratory diseases [19, 20]. Recent data also demonstrated the usefulness of NIV as palliation in different clinical situations, such as patients with “end-stage” solid tumors and ARF, many of them being over seventy-five year’s old [4, 21].

Although NIV could be considered a proper therapeutic tool in elderly population with ARF, some peculiar issues should be considered such as environment, selection of patients, palliative care and “end-of-life” decisions.

#### Environment

**Fig. 2:**
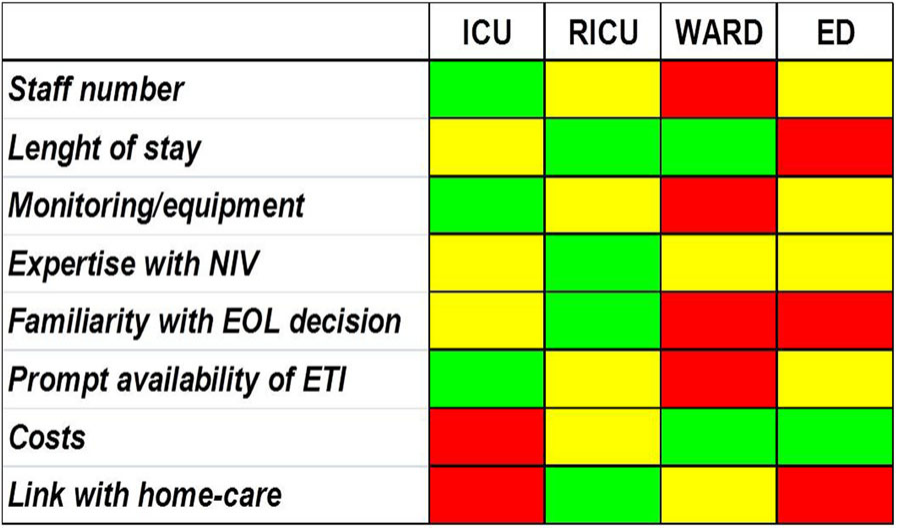
Characteristics of the different settings where non-invasive ventilation (NIV) may be applied. Green, yellow, red indicates respectively a highly favorable, favorable, unfavorable issue for the different enviroment. ICU: Intensive Care Unit, RICU: Respiratory Intensive Care Unit, ED: Emergency Department, ETI: endotracheal intubation, EOL: end-of-life

The most suitable hospital setting where to start NIV in the elderly should have expert staff in an adequate number according to the severity of patient’s conditions for 24/24 cover, multi-parametric monitoring, prompt availability to invasive ventilation, reasonable costs, a structured discharge plan, consideration of “end-of-life” choices (Fig. 2) [10, 22, 23]. ICU fits all these criteria expect for the costs and, therefore, there is an imbalance between the ICU beds and the number of patients requiring NIV. This is particularly true for elderly patients. Starting NIV outside ICU has the advantage of treating less severe patients with the similar rate of success but at lower costs than in ICU [22] avoiding a potential distressing experience. However, the low level of care provided in some environment might increase the risk that deterioration of patients receiving NIV will not be promptly recognized and treated [24]. RICU represents a balanced setting where there are expertise in NIV, cost-effectiveness, knowledge of the history of chronic respiratory patients, awareness of end-of-life issues [25–27].

The term RICU refers to specialized units for patients who require an “intermediate” level of care between the ICU and the ward where non-invasive monitoring and assisted ventilation techniques are mainly, even if not exclusively, applied. RICU acts as “step-down unit” for stabilized patients transferred from ICU (i.e.*,* weaning and decannulation) and as “step-up unit” for cases not responding to medical therapy in emergency departments or wards [25, 27]. As a matter of the fact, the availability of an “intermediate-respiratory” setting to manage “mono-organ” decompensations avoids the dangerous “under-assistance” in low-care environment (i.e.*,* ward) and the uselessly “over-assistance” in high-care environment (i.e.*,* ICU) [25]. This is why RICUs may provide a specialized quality of care for the clinical governance of ARF with health resources optimization (i.e.*,* reduced nurse-to-patient ratio). In fact, compared to the direct and indirect high costs required by traditional ICUs, RICUs advantages are linked to the lower nursing staff requirements and to the improved utilization of the ICU resources (i.e. patients with severe multi-organ failure) [25]. Conversely, discharge from the ICU to the RICU becomes possible for patients who have recovered from the acute phase of critical illness but still need intensive nursing or physiotherapy before being weaned from the ventilator [25]. This concept is particularly true for elderly patients with advanced chronic cardio-pulmonary diseases who are often refused to be admitted to ICU because too old and too ill. Besides the economical factors, there are other advantages which favor the RICU for the management of these patients. More privacy for the patient and easier family’s access which characterize the RICUs may contribute to the “healing” process and facilitate discharge, especially for those patients requiring long-term oxygen therapy and/or mechanical ventilation at home [25]. It should be empathized that the term RICU as “intermediate level of care” has not the same “conceptual meaning” among the different countries and, therefore, a large heterogeneity emerges also within the same country. Basically, the main differences are due to the following factors: human resources (i.e. nurse to patient ratio; availability of physicians 24 h a day; presence or not of respiratory therapists); organization model (i.e. independent RICU vs RICU inside ICU vs RICU inside a ward); specialist vocation (i.e. respiratory vs medical intermediate care unit); functional mission in the Hospital (i.e. step-down unit vs step-up unit vs rehabilitative unit vs weaning unit); practical skills (i.e. management of airway with provision of ETI, extra-pulmonary support) [27].

The issue of where to start NIV is still debated for the heterogeneity of settings capable of delivering NIV even within the same hospital depending on the team’s expertise, the availability of prompt ETI and the existence/lack of well defined step-down and/or step-up pathways [22, 23].

Choice of where starting NIV is based on patient’s need for monitoring, unit’s monitoring capabilities, staff experience, and time-response to NIV [10, 22, 23]. Patients with ARF poorly responsive to NIV, such as pneumonia, ARDS, and asthma, should be treated in ICU, where immediate ETI is available. One exception is when NIV is applied in ‘do-not-intubate’/‘do-not-resuscitate’(DNI)/DNR) context, to palliate symptoms [4, 5]. Fast-responding diseases (ie. acute cardiogenic pulmonary oedema) may be appropriately ventilated in short-stay enviroment, such as pre-hospital transport and emergency department [10, 22, 23].

#### Selection of patients

**Fig. 3:**
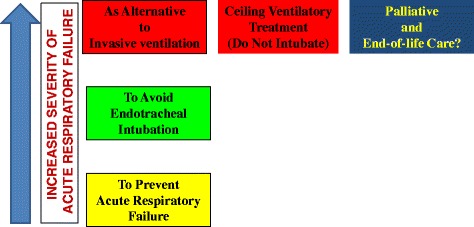
Reasons for applying non-invasive ventilation in elderly at different stages of acute respiratory failure

Concerning this issue some points should be considered when starting NIV in the elderly patients. Figure 3 shows the aims of NIV in the elderly within different clinical scenarios of ARF.

Firstly, clinician ought to have clear in mind the goals to achieve when NIV is applied [4, 5, 8, 28]: 1) to prevent the occurrence of impending (but not established) ARF or post-extubation failure, (2) to prevent further clinical-physiological deterioration and the need for ETI when ARF is already established but ventilatory support is not mandatory; 3) as alternative to IMV when ventilatory support is mandatory *quoad vitam* or as a tool for facilitating the weaning from IMV; 4) as a palliative care in DNI/DNR patients with “end-stage” chronic respiratory or neoplastic diseases. This point strongly influences when and where to start NIV, as well as what to do in case of treatment failure. Concerning the timing, NIV should be started early because a delay may permit further deterioration and increase the likelihood of failure. However, there is no point in starting NIV too early in patients with mild signs of ARF especially in hypercapnic patients [28].

Secondly, clinician has to carefully identify the type of disease in acute decompensation to be treated at first with NIV [10]. A first distinction should be made between hypercapnic ARF mostly occurring in patients with preexistent chronic respiratory disorders (i.e.*,* COPD, chest wall deformities, neuro-myopathies) and hypoxemic or “de novo” ARF occurring in patients without preexistent cardiorespiratory diseases (i.e.*,* ARDS), being the former more responsive to NIV than the latter. An important exception are patients with neuromuscular diseases, particularly progressive conditions such as amyotrophic lateral sclerosis (ALS), that leads to pump failure with progressive hypercapnia; this condition could be effectively managed with NIV provided that bulbar function is preserved [29].

Thirdly, as in any medical interventions, care should be taken in excluding from a NIV trial patients with contraindications**.** Some of these conditions, such as cardio-respiratory arrest, require immediate intubation and invasive ventilation. However, other classical contraindications are derived from exclusion criteria of RCTs and, consequently, it’s more correct to state that NIV is not proven in these circumstances [10, 22]. For instance, NIV is considered contraindicated in encephalopathy based on the concern that it would increase the risk of pulmonary aspiration and reduce patient’s cooperation. This is not true at least for altered level of consciousness due to hypercapnic encephalopathy which may be “safely” reversible with NIV [30]. In a prospective case–control study of patients with COPD exacerbations and moderate-to-severe hypercapnic encephalopathy, most of them in their elderly age, the use of NIV versus IMV was associated with similar short and long-term survival and fewer nosocomial infections [31]. Another example is the feasibility of NIV to successfully treat episodes of ARF occurring in patients having secretion’s retention and/or depressed cough thanks to an integrated strategy aiming at improving airways clearance [14, 32]. While NIV is well tolerated when the sensorium is severely depressed, agitation may ensue when the patient is awaking and prevent him/her to keep on ventilation. A status of agitation and/or delirium frequently occurs in the elderly with ARF. Strategies based on the cautious use of low-doses of sedatives (i.e. opioids) provided in a high level of care setting could be attempted in mildly agitated patients during NIV [33]. In expert hands, patient comfort and patient-ventilator synchrony may be improved by “a safe” sedation even in case of intolerance [32]. Although this strategy is feasible, the risk of oversedation and need for intubation should be carefully considered [10, 34].

The use of predictive factors may be useful in the selection-making process even though those with a greater predictive value (i.e.*,* clinical-physiological parameters after a trial of NIV) are not available before starting ventilation. Nevertheless, the finding at baseline of severe acidosis (i.e. pH < 7,25), remarked “de novo” hypoxemia (i.e. PaO2/FiO2 < 200) and non-pulmonary organ failures are associated with a likelihood of NIV failure [10, 34, 35]. Being considered NIV as the application of “ars medica”, there is not a “magic formula” that precisely will “broadcast” the future of patients started to this “miracle machine” [22]. This is particularly true for elderly subjects with ARF. As a matter of the fact, at the end, whether clinician should attempt NIV is based on individual circumstances. For instance, if IMV is not considered appropriate (i.e.*,* DNI/DNR status), a trial of NIV could be considered. Although there are few contra-indications to NIV in patients deemed not for intubation, the likelihood of a favorable outcome should be discussed with the patients and their families. The reason is why NIV could be an “intrusive therapy”, and if there is little chance of a good outcome initiating NIV may increase distress and alternative palliation strategy would be more appropriate. Table 1 summarizes the likely outcome of NIV for the main clinical indications in the elderly with ARF.

**Table 1:** Likelihood of success of NIV in elderly patients according to the different types of ARF

Clinical condition with Acute Respiratory Failure	Avoidance of intubation Reduction of Mortality
COPD exacerbations	+++
Cardiogenic pulmonary edema	+++
Immuno-compromised status	+++
Obesity-hypoventilation	+++
Chest wall deformities	+++
Weaning/Extubation in COPD	++−
Mild-moderate Encephalopathy	++−
Neuromuscular diseases^a^	++−
Community Acquired Pneumonia	+−−
Mild ARDS	+−−
Agitation/Delirium^b^	+−−
Interstitial fibrotic lung diseases	---
Multi-organ failure/Comorbidities	---

^a^ with cough augmentation techniques

^b^ with low doses of sedatives

### Integrated strategy to avoid NIV failure

#### NIV failure

Even in expert hands, NIV failure may occur in 5–60% of the treated cases, depending on numerous factors, included the severity of ARF, the expertise of the team, and the intensity of care provided by the environment [34]. The early identification of NIV failure is of pivotal importance as the unduly delay of IMV may be associated with an increased mortality. According to the timing of occurrence, NIV failure may be distinguished in: 1) immediate failure (within minutes to <1 h), due to weak cough reflex, excessive secretions, hypercapnic encephalopathy syndrome, intolerance, agitation, and patient-ventilator asynchrony; 2) early failure (from 1 to 48 h), due to poor arterial blood gas and an the inability to promptly correct them, increased severity of illness, and the persistence of a high respiratory rate with respiratory muscle’s distress; and 3) late failure (after 48 h), which can occur after an initial favorable response to NIV and may be related to sleep disturbance and severe comorbidities [34, 35].

#### Integrated strategy

**Fig. 4:**
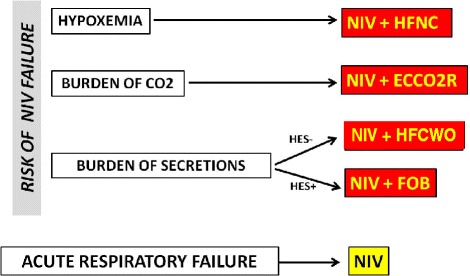
Flow-chart depicting the potential integrated respiratory therapies in adjunct to NIV in case of risk of failure in cases of elderly patients with DNI status. ARF: acute respiratory failure, ECCO_2_R: extracorporeal CO_2_ removal, FOB: flexible bronchoscopy, HES: hypercapnic encephalopathy syndrome, HFCWO: high-frequency chest wall oscillation, HFNC: high-flow nasal cannula, NIV: non-invasive ventilation

The integration of NIV with other less invasive supports (e.g. HFNC, mechanical cough assistance devices, FOB with toilette of abundant secretion, low-flow CO_2_-removal systems) could reduce the rate of NIV failure and may be especially feasible in the elderly in which IMV is not desirable [36–46]. Figure 4 details the different strategies of NIV integrated with other procedures according to various clinical situations.

The conventional oxygen-therapy to treat hypoxemic patients shows several drawbacks [9] such as the imprecise estimation of the delivered fraction of inspired O_2_ (FiO_2_) depending on the patient’s breathing pattern, the CO_2_-rebreathing with “reservoir” devices, the subject’s discomfort due to mask poor tolerance and interference with eating, drinking, speaking, insufficient heating and humidification of the administered oxygen, and last but not least the mismatch between the limited amount of the deliverable oxygen flow and the high patient’s inspiratory request. Furthermore, conventional oxygen therapy is unable of unloading respiratory muscles and may cause a rise in PaCO_2_ level with a contextual drop in pH leading to the need for mechanical ventilation especially in patients with acute on-chronic hypercapnic respiratory failure. HFNC is a new system that is able to deliver up to 100% heated and humidified oxygen at a maximum flow of 60 L/min of gas via a nasal cannula [9]. Thanks to its technological peculiarity, HFNC has several physiological advantages over conventional oxygen therapy: 1) capability of administering precise values of FiO_2_ ranging from 21 to 100%; 2) efficient clearance of CO_2_ correlated with high flushing of pharyngeal dead; 3) good efficiency in humidifying and heating the delivered oxygen-air mixture with an improved capacity of removing secretions; 4) great patient’s comfort towards the treatment which does not interfere with eating, drinking, speaking; 5) adequate matching between the flow rate provided by the device and the patient’s inspiratory demand; 6) “stenting effect” on upper airways and alveolar recruitment due to the generation of flow-dependent low PEEP levels (up to a median of 7.4 cmH_2_O at 60 L/min) [9]. An increasing amount of clinical data, even if mostly uncontrolled, are accumulating about the feasibility, efficacy and tolerance of HFNC in hypoxemic ARF of different etiology with the aims of reducing the escalating ventilatory therapy (i.e.*,* NIV and IMV), in “DNI patients” as alternative to NIV, in “end-stage” chronic cardio-pulmonary diseases with ARF, in post-cardiac surgery patients as prophylactic support to reduce the need of mechanical ventilation, during FOB in high-risk ARF patients. A large of these clinical applications concerns elderly patients with ARF especially those with “DNI-status” who do not cope with NIV [36]. Recent reviews of the literature report the current knowledge about HFNC, from its mechanisms of action to its effects on outcomes in different clinical situations [37, 38].

Another important drawback for the successful use of NIV in patients with a depress cough reflex is the inefficacy to spontaneously clear airways from an excessive burden of respiratory secretions, this is essentially due to the kinds of interfaces used to deliver NIV, which do not allow direct access into the airways. Consequently, the inability to spontaneously remove respiratory secretions has been considered a contraindication to start NIV in ARF, especially in patients with altered level of consciousness associated with depressed cough [7, 8, 10, 11, 34]. Non-invasive and “mini-invasive” integrated strategies may be attempted to avoid NIV failure due to accumulated secretions into the airways tree.

In ARF of neuromuscular origin in patients with preserved bulbar function a large body of evidence support the combined use of NIV plus MI-E for the management of secretions in patients with a severely impaired cough efficiency [14, 34]. However, very recently, a RCT has shown that the “breath-stacking” technique using a lung volume recruitment bag turns out to be as efficient as the MI-E technique in enhancing secretion clearance in ALS patients with ARF [39]. So far, the “breath-stacking” technique could be considered as a low-cost, first-line intervention for volume recruitment and cough augmentation in patients with ALS who meet the criteria for intervention with NIV.

Conversely, few published data suggested that some non-invasive physiotherapeutic techniques may improve mucous clearance in chronic lung diseases in acute exacerbations managed with NIV. Some papers demonstrated that acute-on-chronic respiratory patients with bronchial hypersecretions of different etiology showed improvement of PaO_2_ and PaCO_2_ values when NIV was associated with HFCWO applications [40, 41]. However, a systematic review has not demonstrated the usefulness of HFCWO in improving the clinical outcomes of hospitalized COPD patients in acute exacerbations in comparison with the usual medical care; consequently, the role of this technique in these patients remains still controversial [42]. This is true also for patients with neuromuscular diseases for whom the available data do not support the routine application of HFCWO.

Concerning the role of “therapeutic” use of FOB during NIV [34, 43, 44], in a matched case–control study Scala et al. [16] compared 15 acutely decompensated COPD patients with copious secretion retention and hypercapnic encephalopathy due to pneumonia undergoing early FBO plus BAL during NIV in an expert RICU with 15 controls receiving IMV in the ICU. Two hours of NIV plus FBO significantly improved blood gases, sensorium and cough efficiency without major complications (cardiovascular events, emergent ETI, pneumothorax). Improvement in PaCO_2_ and pH, as well as hospital mortality, and durations of hospitalisation and ventilation were similar in NIV vs IMV groups. NIV significantly reduced serious infectious complications compared with IMV, as well as the need for tracheostomy. Even if this NIV strategy may be a successful alternative to IMV to manage selected COPD patients within expert units, larger RCTs are necessary to confirm this preliminary result.

ECCO_2_R, which developed from the traditional ECMO [17], has been recently proposed as an alternative or an integrated therapeutic option in patients with acute hypercapnic acidotic respiratory failure who are “non-responder” to a NIV trial [45, 46]. While ECMO is a “total extra-corporeal support” which is able to oxygenate severely hypoxemic patients and remove up to 50% of the total body CO_2_ production, ECCO_2_R works as a “partial extracorporeal support” capable of removing lower amount of CO_2_ without substantial effects on the oxygenation. Being less invasive than ECMO (lower blood flows, lower diameter cannulation, lower doses of heparin), ECCO_2_R is associated with fewer severe complications [17]. As well as in ARDS patients and in severely chronically ill patients as bridge to transplant, ECCO_2_R has been recently applied in severe hypercapnic respiratory failure due to COPD exacerbations, mostly occurring in elderly subjects [17, 46]. In a recent matched study with historical controls the addition of ECCO_2_R to NIV in 25 COPD elderly patients with severe acidotic exacerbation at risk of NIV failure was associated with a significant improvement in blood gases and respiratory rate with a non-significant reduction in ETI rate as compared to an matched control group of 21 patients receiving only non-invasive ventilator support [47]. However, 13/25 patients (52%) submitted to ECCO_2_R plus NIV experienced adverse events related to extracorporeal CO_2_ removal. Bleeding episodes were observed in three patients, and one patient experienced vein perforation. Malfunctioning of the system caused all other adverse events. The Authors concluded that even if a short treatment with ECCO_2_R may be efficacy in quickly and persistently removing the excess of CO_2_ in severe hypercapnic acutely decompensated COPD patients who are going to fail with NIV assistance, the widespread use of this approach, especially in DNI elderly patients, could not be recommended due to the potential serious complications and the lack of robust proven clinical evidence. A subsequent systematic review analyzing the effectiveness and safety of ECCO_2_R to avoid intubation or reduce length of invasive ventilation in hypercapnic respiratory failure due to COPD exacerbations highlights that this technique is still experimental and no randomized trial is available. Therefore, higher-quality studies are required to better elucidate the risk-benefit balance of ECCO_2_R [46]. This is particularly true in fragile elderly patients who are at higher risks of hemorrhagic complications.

### NIV and palliation

In case of use of NIV in elderly patients with “DNI-status” having poorly responsive diseases (e.g. “hypoxemia de novo” or interstitial lung diseases) physicians must carefully evaluate the relationship between its potential favorable clinical effects and its risk of uselessly prolongation of a discomfort procedure [4, 5, 19, 26].

The “palliative use” of NIV in patients who have decided to forego ETI and in those with “end-stage” respiratory disease is still controversial according to the available published data [4, 5, 19]. Some Authors have suggested the palliative use of NIV in this scenario to alleviate respiratory distress and/or to allow the communication and/or to provide additional time to finalize personal affairs and to come to the acceptance of death [48]. Conversely, other Authors considered this use inappropriate as NIV is still a form of life support even if delivered non-invasively by a mask that may cause itself discomfort and may prolong uselessly the dying process while diverting critical care resources away from other patients more likely to survive [49]. The more controversial point is whether the benefit of NIV in palliate dyspnea may be outweighed by the discomfort and the limited communication induced by a tight-fitting face mask. In addition to that the physician should not forget to consider and to let the patient/family known the other possible complications of NIV, such as gastro-distension, eye irritation, pneumothorax, agitation, patient-ventilator asynchrony, hemodynamic instability that may further deteriorate the poor quality of life of DNI patients [4, 50, 51].

Recently, a Task Force on the “Palliation Use of NIV” of the Society of Critical Care Medicine [5] suggested to classify the use of NIV for patients with ARF into three categories: 1) NIV as life support with no preset limitations on life-sustaining treatments; 2) NIV as life support when patients and families have decided to forego ETI; 3) NIV as a palliative measure when patients and families have chosen to forego all life support, receiving only comfort measures. This Task Force suggests an approach to use of NIV for patients and families who choose to forego ETI. NIV should be applied after careful discussion of the goals of care, with explicit parameters for likelihood of success and failure, by experienced personnel, and in appropriate healthcare settings. It’s important to acknowledge that individual patients may transition from one to another category as the goals of the care or the risk/benefit balance of NIV may dynamically changed [4, 5].

The goals and the time for discontinuation of NIV are similar for the category of patients who decline ETI and IMV with the difference that NIV will be withdrawn and comfort measure only intensified if NIV is not successful and/or not tolerated any longer. Conversely from the first two categories of candidates for NIV, patients belonging to the third category, such as those at the “end-stage” of a chronically progressive diseases (i.e.*,* COPD, neuromuscular disorders, chronic heart failure) or those with terminal malignancy, most of them over 70 year old, do not want any form of life-prolonging therapy as their baseline quality of life is found unacceptable despite a maximal out-patient therapy. Patients in this category should not be encouraged to tolerate the NIV-associated discomfort because the goal of the chosen therapy is only the palliation of the symptoms and not the improvement of physiological parameters [52]. In this scenario there is no sense in providing NIV to patients who are unable to communicate (i.e.*,* decreased level of consciousness) as they could not feel the potential impact of NIV on their symptoms [4, 5]. This palliative use of NIV may also allow comfort measure only for patients to be transferred home in order to spend the end of his life in his own bed [4, 48, 49]. This scenario is often referred to elderly patients.

Another unexplored question is whether NIV is more effective than pharmacological therapies, such as opiates, in palliating symptoms. It’s a crucial point that the patient can and must keep the control over the decision to carry on NIV. If discomfort related to the use of the mask exceed the benefit the patient may simply choose to discontinue NIV and his/her comfort should be achieved with drugs. The use of anticipated doses of opiates before withdrawing NIV at the end of life may be an option to achieve the higher level of patient comfort, similarly to what was already reported with invasive mechanical ventilation [53]. The transition from mechanical support to an oxygen mask looks much simpler both ethically and technically with NIV than with invasive ventilation.

A very appealing goal of NIV in such kind of patients is to achieve a good control of the dyspnea in addition to the traditional pharmacological therapy. Results of a recent multicenter randomized controlled study [21] performed in advanced solid cancer patients showed that compared to the only oxygen and medical therapy the adjunct of NIV may reduce the amount of the needed doses of opiates and therefore their side effects, such as the depressed level of sensorium. Thus may mean a better capability of communication for the patient at the end of his life with a good control of symptoms.

## Conclusions

Treatment of ARF in the elderly should take into account the technical and scientific aspects of ventilatory and non-ventilatory supporting devices as well as ethical and economic issues that have to be contextualized depending on the type of disease, the degree of patient information, the achievement of shared choices, local health resources, team expertise in mechanical ventilation. NIV is the ventilatory support of first choice in the elderly when physician considers the correct selection of the case and the appropriate choice of the setting and of the timing of application. NIV failure should be managed carefully depending on the will of the patients to undergo either to the escalating therapy or the palliative care.

## Abbreviations

ALS: Amyotrophic lateral sclerosis
ARDS: Acute respiratory distress syndrome
ARF: Acute respiratory failure
COPD: Chronic obstructive lung disease
DNI: Do-not intubate
DNR: Do not resuscitate
ECCO_2_R: Extracorporeal CO_2_ removal
ECMO: Extra-corporeal membrane oxygenation
ETI: Endotracheal intubation
FiO_2_: Fraction of inspired O_2_
FOB: Flexible bronchoscopy
HFCWO: High frequency chest wall oscillation
HFNC: High-flow nasal cannula
ICU: Intensive care unit
IMV: Invasive mechanical ventilation
MI-E: Mechanical insufflator-exsufflator
NIV: Non-invasive ventilation
RICU: Respiratory intensive care unit
